# A Successful Case Report of Epipharyngeal Abrasive Therapy (EAT) in a Patient With Sleep Apnea Syndrome and Traumatic Tympanic Membrane Perforation Caused by Chronic Epipharyngitis

**DOI:** 10.7759/cureus.59089

**Published:** 2024-04-26

**Authors:** Ito Hirobumi

**Affiliations:** 1 Otolaryngology, Ito ENT Clinic, Funabashi, JPN

**Keywords:** eat, airway resistance in the epipharynx, mucociliary secretory system, autonomic function, eustachian tube dysfunction, epipharyngeal abrasive therapy, chronic epipharyngitis, sleep apnea syndrome, traumatic tympanic membrane perforation

## Abstract

Traumatic perforation of the tympanic membrane often occurs in Japanese patients who scratch their ears to relieve itching. Traumatic tympanic membrane perforation may close spontaneously, but the perforation may remain. One of the causes of prolonged tympanic membrane perforation closure is dysfunction of the Eustachian tube. In this study, I experienced a case of chronic epipharyngitis causing ear fullness and itching, and a traumatic perforation of the tympanic membrane caused by scratching with an earpick. The patient also had sleep apnea syndrome (SAS). Treatment of chronic epipharyngitis with epipharyngeal abrasive therapy (EAT) shortened the time to perforation closure and improved SAS, suggesting that EAT affected the improvement of Eustachian tube function and airway resistance in the epipharynx.

## Introduction

In Japan, traumatic perforation of the tympanic membrane is a common cause of itchy ears due to ear scratching [1]. Traumatic tympanic membrane perforation may close spontaneously, but in some cases, the perforation remains and requires surgical treatment [2]. One of the causes of prolonged perforation of the tympanic membrane is the size of the perforation. The larger the perforation, the longer it takes to close [3]. Eustachian tube dysfunction can also cause infections such as acute otitis media, leading to prolonged perforation of the tympanic membrane [4]. Chronic epipharyngitis can be broadly classified into three groups: (1) related symptoms due to local inflammation, (2) systemic symptoms due to autonomic and endocrine disorders, and (3) diseases and symptoms mediated by autoimmune mechanisms. Chronic epipharyngitis is thought to be a combination of these pathologies, resulting in a variety of symptoms. The vagus nerve is distributed in the epipharynx and external auditory canal. In this case, chronic epipharyngitis induced itching of the ears via Arnold's nerve reflex, and inflammation and swelling of the epipharyngeal mucosa disrupted Eustachian tube function and induced ear fullness. Ear scraping resulted in traumatic perforation of the tympanic membrane. In addition, chronic epipharyngitis may have increased airway resistance in the nasopharynx, resulting in sleep apnea. The tympanic membrane perforation was large and hearing loss was significant, but epipharyngeal abrasive therapy (EAT) for chronic epipharyngitis resulted in the closure of the tympanic membrane perforation and improvement of hearing loss in a short period of time. EAT also improved the previously observed sleep apnea syndrome (SAS). It is thought that EAT improved Eustachian tube function and accelerated healing of the perforated tympanic membrane. EAT may also improve SAS by decreasing airway resistance in the nasopharynx. This paper discusses the literature review of the effects of EAT on Eustachian tube function and airway resistance in the nasopharynx and reports that EAT may be a potential treatment modality to improve Eustachian tube dysfunction and SAS.

## Case presentation

**Figure 1:**
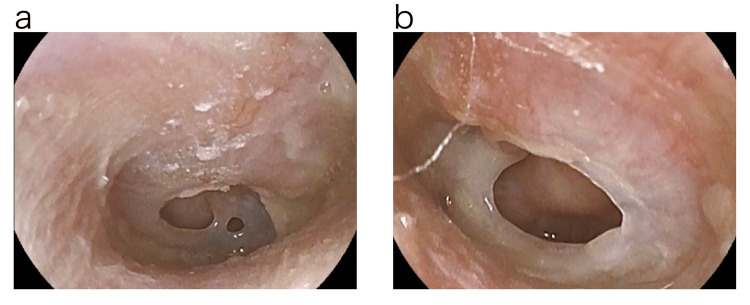
Photograph of eardrum before treatment. Two central tympanic membrane perforations centered in the anterior lower quadrant were observed in the right ear. The left ear showed a large central tympanic membrane perforation of more than 3 mm in diameter extending from the anterior upper quadrant to the anterior and posterior lower quadrants. a: Right tympanic membrane. b: Left tympanic membrane.

A 28-year-old female presented to our clinic in February 2018, with a chief complaint of ear fullness and itching in both ears for about a week. With the diagnosis of ear canal eczema, betamethasone valerate/gentamicin sulfate ointment was prescribed. About two weeks later, the patient developed hearing loss after she rubbed the ear canal strongly with a cotton swab by herself. The next day, the patient visited our clinic due to the appearance of ear leakage. On examination, two central tympanic membrane perforations centered in the anterior lower quadrant were observed in the right ear. In the left ear, a large central tympanic membrane perforation of more than 3 mm in diameter was observed extending from the anterior upper quadrant to the anterior and posterior lower quadrants. Bilateral tympanic membrane perforations were thought to have occurred simultaneously (Figure 1).

**Figure 2:**
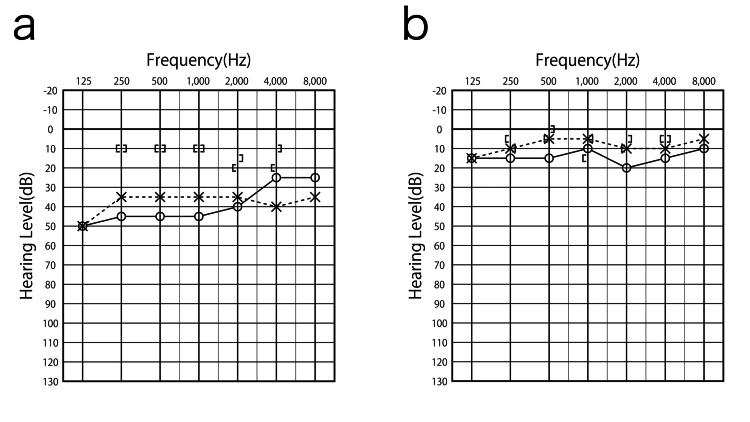
Audiogram before and after treatment. The patient had a conductive hearing loss of 43.8 dB in the right ear and 35.0 dB in the left ear (quadrant, mean hearing level). The size of the tympanic membrane perforation was smaller in the right ear than in the left ear, but the hearing loss was greater in the right ear. After treatment, hearing levels improved to 13.8 dB in the right ear and 6.3 dB in the left ear. a: Audiogram before treatment. b: Audiogram after treatment.

Standard pure tone audiometry (Morita Corporation audiometer component system SA1-60, Osaka, Japan) revealed a conductive hearing loss of 43.8 dB in the right ear and 35.0 dB in the left ear (quadrant, average hearing level). The size of the tympanic membrane perforation was smaller in the right ear than in the left ear, but the hearing loss was greater in the right ear (Figure 2).

The diagnosis of bilateral tympanic membrane perforation was made due to ear-piercing trauma. The ear leaks from the perforated eardrum were suctioned out and the ear was cleaned. Ofloxacin otic solution 0.3% ear drops were used.

The patient had a history of snoring and daytime sleepiness for several years but had neglected these symptoms until now without undergoing any specific examination or treatment. The patient was thin and no obesity was observed. There was no evidence of nasal septal curvature. The patient had a habit of scratching the ears because of a feeling of ear closure and itching when the abnormal sensation in the epipharynx became stronger. There were no family history items of note.

**Figure 3:**
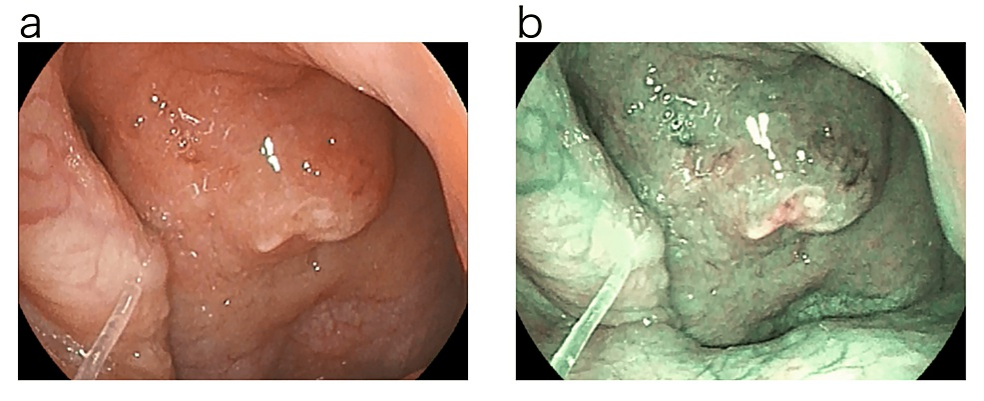
Endoscopic findings of the epipharynx before treatment. Examination using normal light revealed swelling of the epipharyngeal mucosa centered on the Thornwald site and an accumulation of an abscess. Examination using optical enhancement mode 1, which uses band-limited light, revealed reddish-brown or dark-brown findings on the mucosal surface that may indicate internal hemorrhage. The deep mucosa showed a greenish color, which may indicate deep vascular dilatation or congestion. a: Normal light. b: Optical Enhancement Mode 1.

Since it was thought that the epipharyngeal lesion might have triggered the onset of traumatic perforation of the tympanic membrane, the epipharynx was examined four days later with a band-limited optical nasopharyngoscopy system (Pentax EPK-i7000 video processor, VNL11-J10 video scope with a 3.5 mm outer diameter tip, Pentax Medical, Tokyo, Japan). Examination using normal light revealed swelling of the epipharyngeal mucosa centered at the Thornwald site and an accumulation of an abscess. The examination using Optical Enhancement Mode 1. with band-limited light showed reddish-brown or dark-brown findings on the mucosal surface, which may indicate internal hemorrhage. The deep mucosa showed a greenish color, which may indicate deep vascular dilatation or congestion (Figure 3) [5].

**Figure 4:**
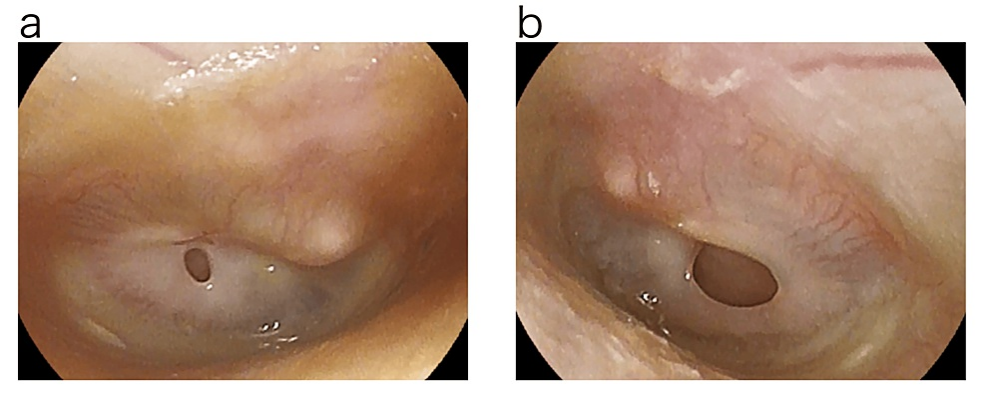
Photograph of eardrum during treatment. The tympanic membrane perforation gradually shrank after epipharyngeal abrasive therapy (EAT). a: Right tympanic membrane. b: Left tympanic membrane.

**Figure 5:**
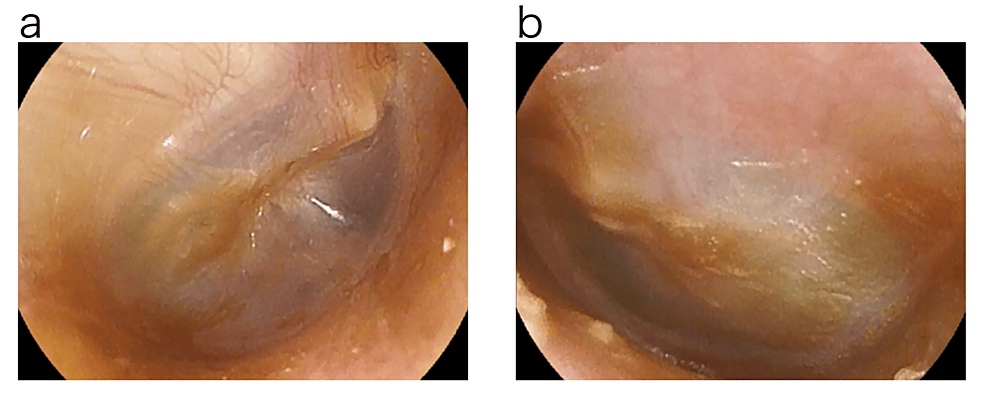
Photograph of eardrum after treatment. Epipharyngeal abrasive therapy (EAT) resulted in the closure of bilateral tympanic membrane perforations in a short period of time. a: Right tympanic membrane. b: Left tympanic membrane.

EAT was performed as a diagnostic treatment; EAT consisted of nasal abrasion treatment with a Lutze swab soaked in 1% zinc chloride solution followed by oral abrasion treatment with a Zermat's pharyngeal crimp cotton swab. When zinc chloride solution was applied to the epipharyngeal mucosa, bleeding was observed and pain was perceived. According to Tanaka's diagnostic criteria, the patient was diagnosed with chronic epipharyngitis [5]. For symptoms such as snoring and daytime sleepiness, a Simple Sleep Apnea Test was performed at bedtime using a sleep evaluation device (Smart WatchPMP-300E by Pacific Medico, Tokyo, Japan). As a result, the Respiratory Disturbance Index (RDI) was 5.5, and a diagnosis of SAS was made [6]. The tympanic membrane perforation was not directly closed, but EAT was performed for chronic epipharyngitis. The size of the tympanic membrane perforation was gradually reduced by EAT (Figure 4), and closure of the perforation was confirmed at the examination about two weeks later (Figure 5).

**Figure 6:**
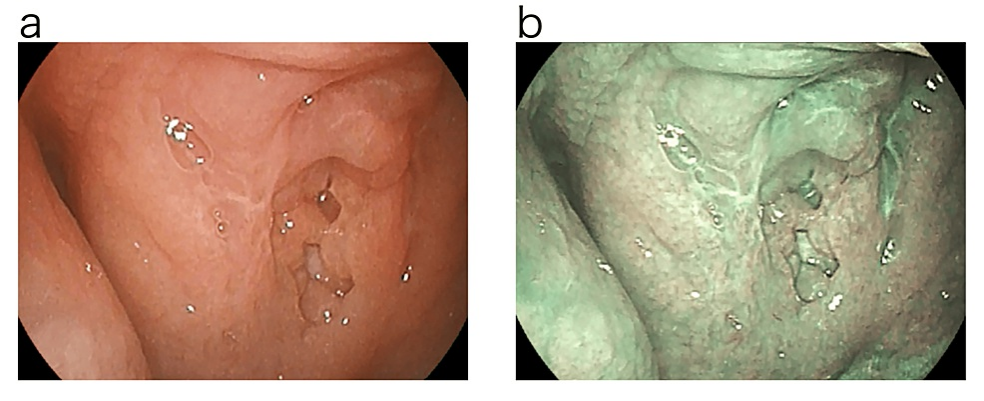
Endoscopic findings of the epipharynx after treatment. The swelling of the epipharyngeal mucosa and the presence of thick plugs improved after epipharyngeal abrasive therapy (EAT), and the bleeding observed during EAT also disappeared. a: Normal light. b: Optical Enhancement Mode 1.

The tympanic membrane perforation was closed after four EAT sessions over a period of about two weeks. EAT was also continued, as EAT improved the symptoms of otorrhea, itching, and SAS. The RDI improved to 3.2 on the Simple Sleep Apnea Test performed approximately one month later. About three months after the traumatic perforation of the tympanic membrane, subjective symptoms such as a sense of ear closure, itchy ears, and SAS symptoms disappeared, swelling of the epipharyngeal mucosa and the finding of thick plug adhesion also improved, and the finding of bleeding during EAT also disappeared, so the chronic epipharyngitis was judged to be cured (Figure 6).

During this treatment period, EAT was performed a total of 13 times, which means that EAT was performed about once a week. The tympanogram recovered to type A, and hearing levels improved to 13.8 dB in the right ear and 6.3 dB in the left ear (Figure 2).

## Discussion

This case of chronic epipharyngitis treated with EAT may have accelerated healing of traumatic tympanic perforation and improved SAS, and the mechanisms by which EAT affects traumatic tympanic perforation and SAS are discussed in the literature and reported here.

Since Japanese people have a habit of cleaning their ears with earpicks and cotton swabs, perforation of the tympanic membrane due to traumatic ear perforation has been reported frequently [2]. Traumatic perforation of the tympanic membrane is closed with conservative treatment in 69.4% to 90.3% (average 80%), but an average of 20% of tympanic membrane perforations do not close with conservative treatment. The time to closure is reported to be longer the larger the perforation [7]. According to the classification by Yoshikawa et al., the size of the tympanic membrane perforation in this case was Grade II in the right ear and Grade III in the left ear, which is considered a large perforation [7]. According to Yamazaki et al., the average time to perforation closure was reported to be 48.4 days for Grade II and 90 days for Grade III [2]. If there are no serious complications such as damage to the ear ossicles or exolymphatic leakage, a policy of no treatment and observation may be adopted, but procedures such as cauterization of the perforation margins or patching may also be performed. Tachibana et al. observed 40 patients with traumatic tympanic membrane perforation over a long period of six months [8]. Of these, 27 (67%) closed spontaneously, 15 (55%) within one month, five (18.5%) within three months, and three (11.1%) within six months. However, four cases (14.8%) closed after more than six months. The authors reported that spontaneous closure of tympanic membrane perforation was prolonged when the perforation margin was in contact with the malleus [8].

Sood et al. measured the size of tympanic perforation and classified small perforation (0-9 mm^2^) as group I [9]. Medium perforation (9-30 mm^2^) was classified as group II. Large perforation (>30 mm^2^) was classified as group III. The mean surface area of the intact tympanic membrane was 64.3 mm^2^. As a result, the mean hearing loss of group I was reported as 31.42 ± 7.15 dB, group II as 39.42 ± 8.97 dB, and group III as 48.91 ± 7.38 dB [9]. The hearing loss level of the right ear in the present case was 43.8 dB and was considered to fall into groups II to III, while the left ear was 35.0 dB and was considered to fall into groups I to II. The right ear had significant hearing loss but improved to 13.8 dB. Despite the size of the tympanic membrane perforation and the significant hearing loss, the perforation was closed within about two weeks after the injury by EAT.

Aging, Eustachian tube dysfunction, and inflammation of the epipharynx have been reported as factors that cause prolonged perforation closure [7,9,10]. In the present case, treatment of chronic epipharyngitis with EAT may have improved Eustachian tube function and accelerated the closure of the tympanic membrane perforation. The Eustachian tube has three major functions. The first function is to equalize the pressure between the middle ear cavity and external air pressure, the second is to excrete foreign substances through the excretory action of the Eustachian tube epipharyngeal mucosa, and the third is to protect against infection from the epipharyngeal mucosa. In the presence of chronic epipharyngitis, pressure on the Eustachian tube orifice due to epipharyngeal mucosa swelling and infection is thought to disrupt these three Eustachian tube functions and induce infection of the middle ear cavity. EAT is thought to have improved Eustachian tube function and facilitated the healing of tympanic membrane perforation by treating chronic epipharyngitis.

The first mechanism by which EAT improves Eustachian tube function is thought to be that EAT acts to open the Eustachian tube by removing organic obstacles such as inflammation of the Eustachian tube orifice or pressure from the adenoids. Second, EAT may improve Eustachian tube function by inducing the pharyngeal reflex, which causes contraction of the palatine sail tension muscle and opening of the Eustachian tube pharyngeal opening. Third, EAT has an autonomic nerve-stimulating effect, and repeated treatment with EAT stimulates sympathetic nerve activity [11]. Relative inhibition of parasympathetic nerve activity reduces swelling of the Eustachian tube mucosa, decreases the viscosity of Eustachian tube mucus secretions, and decreases secretions from the Eustachian tube mucosa, which is thought to act to open the Eustachian tube [12]. Eustachian tube function may be improved by the synergistic action of these three effects of EAT.

Eustachian tube dysfunction (ETD) manifests as Eustachian tube stenosis and Eustachian tube opening symptoms [13]. Eustachian tube ventilation therapy is used to treat Eustachian tube stenosis symptoms, but the problem with Eustachian tube ventilation therapy is that it passively opens the Eustachian tube, but the effect is not long-lasting. Treatment methods such as balloon Eustachian tuboplasty (BET) have been tried for refractory ETD, but there are problems with treatment cost, invasiveness, etc. [14]. EAT has the advantage of being noninvasive, simple, and repeatable in an outpatient setting. EAT actively opens the Eustachian tube by inducing the pharyngeal reflex. Repeated stimulation is thought to restore autonomic reflexes and allow spontaneous Eustachian tube opening. EAT may be a useful treatment for ETD.

The patient's RDI improved with EAT, and subjective symptoms of SAS also improved. Continuous positive airway pressure (CPAP) is a treatment for SAS, but it is difficult to cure it. Otolaryngologic surgical procedures that can be expected to cure SAS include uvulopalatopharyngoplasty (UPPP), tonsillectomy, adenotomy, and septorhinoplasty. However, no matter which technique is chosen, it is important to consider the specifics of the procedure, Regardless of the technique chosen, there are advantages and disadvantages to each technique [6]. EAT is thought to improve SAS by treating the inflammation and swelling of the epipharyngeal mucosa caused by chronic epipharyngitis, thereby reducing airway resistance in the epipharynx. EAT may also affect the secretory glands of the epipharyngeal mucosa through autonomic nerve stimulation [11]. EAT may improve the mucus-filial system, lubricate the epipharyngeal mucosa, and improve airway resistance [15]. EAT is a noninvasive, easily repeated treatment in an outpatient setting and may be a useful treatment for SAS. 

This paper is a case report and is limited in its ability to address whether EAT shortens the duration of tympanic membrane perforation closure. It is also unclear whether EAT is useful for traumatic tympanic membrane perforation without chronic epipharyngitis. In this case, treatment of chronic epipharyngitis may have improved Eustachian tube function and shortened the duration of tympanic membrane perforation closure, but the specific effects of EAT on Eustachian tube function remain to be investigated. Although it is commonly experienced in daily practice that EAT improves SAS, an epidemiological investigation of the relationship between chronic epipharyngitis and SAS is needed to collect a larger number of cases. EAT is a noninvasive, simple, and repeatable treatment that can be performed in an outpatient setting, and may be an effective treatment for EDT and SAS in the future. As far as the author could find, there have been no reports on the effects of EAT on EDT or SAS, so this report is presented here.

## Conclusions

A case of traumatic tympanic membrane perforation and SAS was treated with EAT, which facilitated closure of the tympanic membrane perforation and improved SAS. EAT is thought to improve Eustachian tube function by removing inflammation and swelling of the epipharyngeal mucosa and the mucosa surrounding the Eustachian tube orifice. It is also thought to improve airway resistance in the epipharynx. EAT may be a potential treatment for EDT and SAS.
